# Inadequate sleep duration may attenuate the anti-inflammatory effects of fish consumption in a healthy Japanese population: a cross-sectional study

**DOI:** 10.1017/S0007114522002896

**Published:** 2023-06-28

**Authors:** Shigemasa Tani, Kazuhiro Imatake, Yasuyuki Suzuki, Tsukasa Yagi, Atsuhiko Takahashi, Naoya Matsumoto, Yasuo Okumura

**Affiliations:** 1 Department of Health Planning Center, Nihon University Hospital, Tokyo, 1018309, Japan; 2 Department of Cardiology, Nihon University Hospital, Tokyo, Japan; 3 Department of Medicine, Division of Cardiology, Nihon University, School of Medicines, Tokyo, Japan

**Keywords:** Atherosclerotic CVD, Fish consumption, Sleep duration, Leucocytes

## Abstract

High fish consumption may be associated with lower inflammation, suppressing atherosclerotic CVD (ASCVD). Long sleep duration, as well as short sleep, may contribute to inflammation, thus facilitating ASCVD. This study investigated the overall association between fish consumption, sleep duration and leucocytes count. We conducted a cross-sectional study between April 2019 and March 2020 with a cohort of 8947 apparently healthy participants with no history of ASCVD (average age, 46·9 ± 12·3 years and 59 % males). The average frequency of fish consumption and sleep duration were 2·13 ± 1·26 d/week and 6·0 ± 0·97 h/d. Multivariate linear regression analysis revealed that increased fish consumption was an independent determinant of sleep duration (*β* = 0·084, *P* < 0·0001). Additionally, habitual aerobic exercise (*β* = 0·059, *P* < 0·0001) or cigarette smoking (*β* = −0·051, *P* < 0·0001) and homoeostasis model assessment-insulin resistance (HOMA-IR) (*β* = −0·039, *P* = 0·01) were independent determinants of sleep duration. Furthermore, multivariate linear regression analysis identified fish consumption as an independent determinant of leucocytes count (*β* = −0·091, *P* < 0·0001). However, a significant U-shaped curve was found between leucocytes count and sleep duration, with 6–7 h of sleep as the low value (*P* = 0·015). Higher fish consumption may be associated with a lower leucocytes count in the presence of adequate sleep duration and healthy lifestyle behaviors. However, long sleep duration was also related to increased inflammation, even in populations with high fish consumption. Further studies are needed to clarify the causality between these factors.

## Graphical abstract

**Figure:**
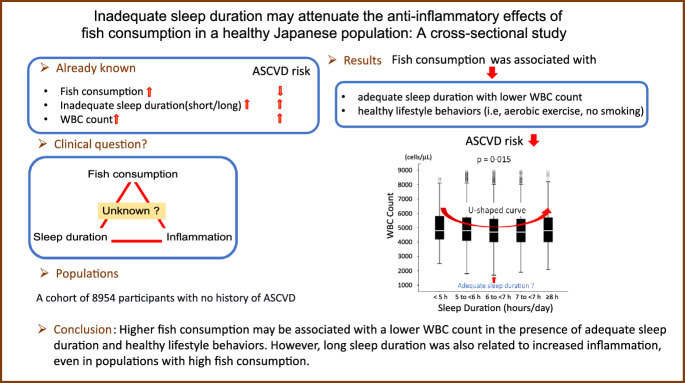

Epidemiological studies have demonstrated the existence of an inverse correlation between the amount of fish consumption and the risk of atherosclerotic CVD (ASCVD)^(1–3)^. The mechanism of suppression of ASCVD by fish consumption largely depends on the cardiovascular protective anti-inflammatory effects of *n*-3 PUFA^(4)^.

Observational studies have indicated that short sleep duration is associated with visceral obesity, glucose intolerance, dyslipidemia and hypertension, resulting in ASCVD^(5)^. Short sleep duration activates systemic inflammation, which increases inflammatory markers, including leucocytes count^(6)^, which may lead to the development of ASCVD^(7)^. In fact, inflammation in the vascular wall plays a crucial role in the advancement of atherosclerosis. From a pathological viewpoint, all stages of the process of atherosclerosis are inflammatory responses to injury^(8)^. Therefore, leucocytes count, a marker of chronic inflammation, plays a vital role in the development of ASCVD^(9)^.

Good lifestyle behaviours, such as healthy dietary habits, are closely associated with a reduced risk of ASCVD^(10)^. In particular, a dietary style involving a high consumption of fish, such as the Japanese dietary style, is also widely recognised to be related to a suppressed risk of the onset of ASCVD^(11)^. In fact, fish consumption has been reported to be positively associated with the intake of other foods that are considered healthy as well as with other healthy lifestyle behaviours (e.g. aerobic exercise habit, lack of cigarette smoking habit and high-quality sleep)^(12,13)^.

Notably, we proved that the EPA:arachidonic acid ratio, a marker of inflammation, derived from daily fish consumption, is an independent predictor of low leucocytes count^(14)^. Recently, we reported that a high frequency of fish consumption by healthy individuals was associated with a low leucocytes count^(15,16)^. However, scarce data exist on the overall relationship among fish consumption, sleep duration and leucocytes count.

We hypothesised that the anti-inflammatory effect of fish consumption on the risk of ASCVD may be associated with sleep duration.

This study aimed to investigate the overall association among habitual fish consumption, sleep duration and leucocytes count, which was used as an indicator of chronic inflammation, in an apparently healthy Japanese population using a cross-sectional study approach.

## Methods

### Study design and sample

Among 11 673 Japanese people who underwent their annual health checkups between April 2019 and March 2020 at the Health Planning Center of Nihon University Hospital, located in the centre of Tokyo, we included 8947 apparently healthy participants in this study.

**Fig. 1.**
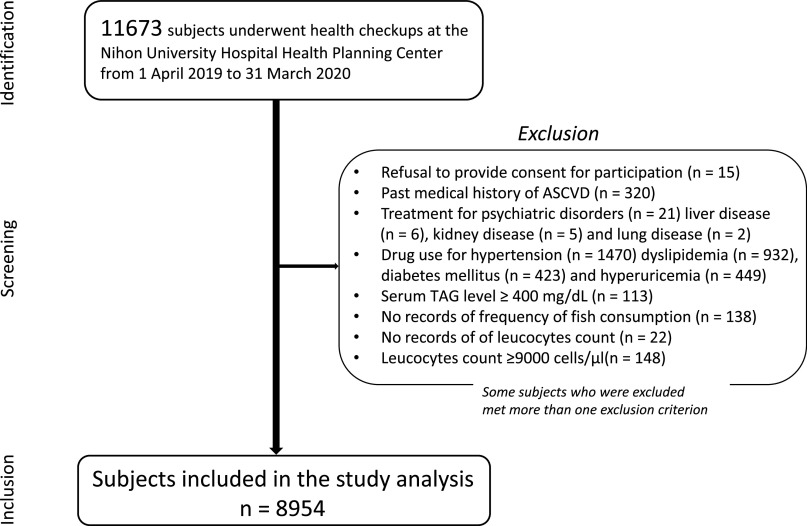
Flow diagram of participant selection. ASCVD, atherosclerotic CVD.

The exclusion criteria were as follows: unwilling to provide consent for participation in the study; positive history of ASCVD; treatment for psychiatric disorders, liver disease, kidney disease and lung disease; current intake of lipid-modifying, antihypertensive, antidiabetic and antihyperuricemic drugs; serum TAG level ≥ 400 mg/dl or absence of data on the frequency of fish consumption and leucocytes count; and blood leucocytes count ≥9000 cells/μl, suggesting the presence of a potentially infectious condition. Fig. 1 shows the flow diagram for the selection of study participants. This study is a sub-analysis of our previous study^(16)^.

This study complied with the Declaration of Helsinki. The study design and objectives were approved by the Institutional Review Board of Nihon University Hospital (approval number: 20200405). The requirement for written informed consent was waived because this was a retrospective cross-sectional study, and an opt-out recruitment procedure was followed. The study was also registered with UMIN (http://www.umin.ac.jp/) Study ID: UMIN 000 043 206.

### Questionnaire on health behaviours

Trained interviewers conducted health behaviour surveys via face-to-face interviews with the participants at our institute. The surveys comprised comprehensive questions to assess the participants’ demographic and socio-economic characteristics, such as age, occupation, marital status, lifestyle behaviours, medication and family history. The individuals undergoing health checkups were administered a lifestyle questionnaire. The questions listed below are similar to the ones used in our previous studies^(17)^.

1. **Smoking habit:** Do you smoke habitually?: No/Yes/I have quit smoking/I quit smoking () years ago.
2. **Drinking habit:** Please specify the frequency of your drinking: Every day/sometimes/I used to drink previously, but have stopped drinking/I stopped drinking () years ago/I drink rarely/I cannot drink; How much do you drink per d when you drink? (ethanol equivalent (g/d)): <20 g/20 to <40 g/40 to <60 g/≥60 g; How many days per week do you drink?^(17)^
3. **Aerobic exercise habit:** Have you engaged in an exercise that makes you sweat slightly for ≥30 min a day, at least twice a week for ≥1 year?
4. **Intensive physical activity:** Do you walk or engage in similar physical activity for 1 h or more per d in daily life?
5. **Sleep habit**: How many hours a day, on average, do you sleep?
6. **Fish consumption**: How many days in a week, on average, have you eaten fish in the past 1 month?

Fish consumption was assessed in weekly frequency in the questionnaire (not at all, once, twice, three times, four times, five times, or six times a week, or approximately every day). The questionnaire is a modified version of the Questionnaire on Specific Health Examination, used for specific health guidance after health checkups under the jurisdiction of the Ministry of Health, Labour and Welfare (MHLW) of Japan^(18)^. The questions listed above are part of a questionnaire that is relevant to this study (online Supplementary Table 1). Furthermore, based on this questionnaire, we excluded participants who met the exclusion criteria at the screening stage (i.e. history of the disease, the disease is being treated, and presence or absence of medications) to determine eligibility for participation in the study.

### Assessment of the estimated weekly average amount of fish consumption

The National Health and Nutrition Survey of Japan estimated the average daily amount of fish consumption according to age group by estimating the ‘net food supply per person per year of fishery products for human consumption’ based on domestic fish production, imports and exports, changes in stocks, and population among others. These data were obtained from the survey conducted by the MHLW of Japan (online Supplementary Table 2)^(19,20)^. Based on this survey record, we calculated the estimated average weekly amount of fish consumption as follows:

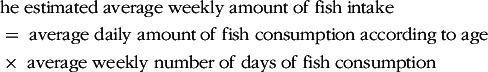




Accordingly, we divided the average weekly amount of fish consumption into seven categorical variables: (1) <50 g/week; (2) ≥50 g/week but <100 g/week; (3) ≥100 g/week but <150 g/week; (4) ≥150 g/week but <200 g/week; (5) ≥200 g/week but <250 g/week; (6) ≥250 g/week but <300 g/week and (7) ≥300 g/week. We have already proven the validity of the estimated fish intake formula in our previous study^(16)^.

### Health examinations and blood samples

We measured the anthropometric variables (i.e. height, weight and waist circumference) of the participants in the standing position, using standardised techniques and equipment. We calculated the BMI by dividing the body weight (kg) by height squared (kg/m^2^) and the waist circumference, using a non-stretchable tape around the participants’ umbilicus in the late exhalation phase^(21)^. We measured the blood pressure twice, with a 3-min interval between the two measurements, using a standard mercury sphygmomanometer after a 5-min rest period; we used the average of the first and second measurements for our assessment. Fasting blood samples were collected early in the morning after the participants had fasted for 8 h. The leucocytes count was determined using a Beckman Coulter STKS (Beckman Coulter, Fullerton, CA). The serum C-reactive protein level was measured by a nephelometric assay (Behring Diagnostic). The estimated glomerular filtration rate was calculated using the abbreviated MDRD (Modification of Diet in Renal Disease) study formula modified by a Japanese coefficient^(22)^. The serum total cholesterol, TAG and HDL-cholesterol levels were measured using enzymatic methods. Furthermore, the Friedewald formula was used to estimate the serum level of LDL^(23)^, and the serum level of non-HDL-cholesterol was calculated by subtracting the serum HDL-cholesterol from the serum total cholesterol. The Hb A1c value was measured by HPLC. Notably, the homoeostasis model assessment-insulin resistance (HOMA-IR), or the insulin resistance score, was calculated as fasting insulin level (mU/ml) × fasting blood glucose level (mg/dl)/405.

### Statistical analysis

Data were expressed as means and standard deviation for continuous variables and as percentages for discrete variables concerning participant characteristics. For cases showing significantly skewed distribution, the data were expressed as the median and interquartile range (IQR). Through ANOVA, continuous variables were compared according to sleep duration as five categorical variables (<5 h, 5–6 h, 6–7 h, 7–8 h and ≥ 8 h). We used the Kruskal–Wallis test for non-parametric multigroup comparison. We subsequently performed a *χ* ^2^ test to compare categorical variables. To investigate the overall relationship between fish consumption, sleep duration and leucocytes count, we examined four items as follows, multilaterally: (1) characteristics of study participants, including fish consumption according to sleep duration; (2) factors influencing sleep duration; (3) association of sleep duration and leucocytes count with fish consumption; and (4) relationship between sleep duration and leucocytes count. Furthermore, we performed a univariate linear regression analysis using sleep duration as the dependent variable, and participant characteristics, ASCVD risk factors, and lifestyle behaviours, including the weekly fish consumption, as independent variables. Determinants deemed significant via a univariate linear regression analysis with *P* < 0·05 were entered into the multivariate linear regression analysis model. Regression analysis was performed using linear regression and Spearman’s and Pearson’s correlation coefficients. We also performed univariate and multivariate linear regression analyses using the study participants’ leucocytes count as the independent variable and fish consumption and participant characteristics as the dependent variables. Habitual fish consumption is reported to be positively associated with healthy lifestyle behaviours (e.g. aerobic exercise and lack of a smoking habit)^(12,13)^. Thus, two-way ANOVA (interaction test) was used to confirm the interaction between the amount of fish consumption and lifestyle behaviours, which were independent determinants of sleep duration in univariate and multivariate linear regression analysis. Similarly, to assess whether fish consumption and sleep duration interact with the leucocytes count, we performed a two-way ANOVA with leucocytes count as the independent variable and fish consumption and sleep duration as the dependent variables. We also performed Jonckheere–Terpstra and Mantel–Haenszel trend tests. All statistical analyses were performed using SPSS software (IBM) for Windows (version 24).

## Results

### Participant characteristics

**Fig. 2.**
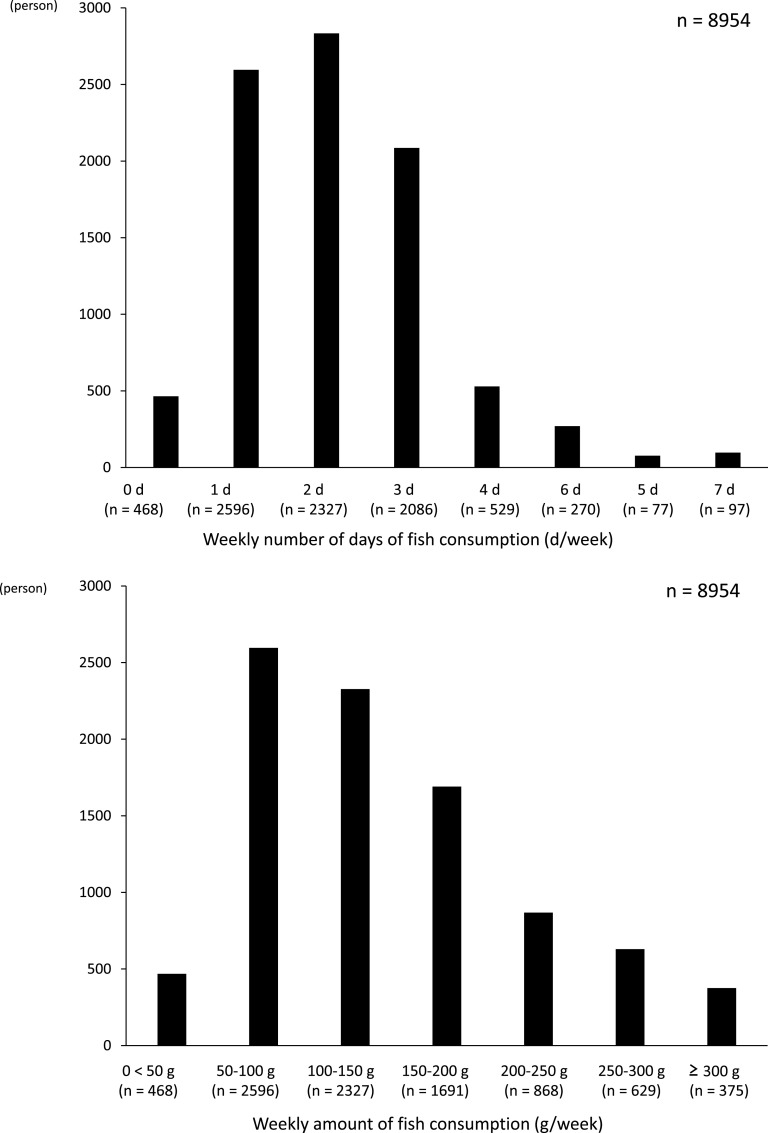
Frequency of distribution of weekly fish consumption.

The average daily sleep duration in the total population was 6·0 ± 0·97 h/d (range: 1–12 h/d). The sex composition of this study included 59 % males (*n* 5278) and 41 % females (*n* 3676). The average age of the 8954 participants was 46·9 ± 12·3 years (males: 48·5 ± 12·7 years (range: 18–89 years), females: 44·6 ± 13·0 years (range: 19–92 years)). Table 1 shows the demographic and participant characteristics in the entire population and across sleep duration categories. Fig. 2 shows the frequency of distribution of the weekly fish consumption. The median (IQR) weekly amount of fish consumption was 111 (67/254) g (range: 0–592 g). The average frequency of fish intake was 2·14 ± 1·28 d/week.

**Table 1.** Comparison of participants’ characteristics, ASCVD risk and lifestyle behaviours according to sleep duration

			Sleep duration (h/d)										*P*
			<5 h		5 to <6 h		6 to <7 h		7 to <8 h		≥8 h		
	All cases *n* 8954		*n* 424		*n* 2074		*n* 3772		*n* 2179		*n* 505		
	*n*	%	*n*	%	*n*	%	*n*	%	*n*	%	*n*	%	
% of participants	100		4·74		23·16		42·13		24·34		5·64		
Male sex	5278	58·9	264	62·3	1298	58·2	2223	58·9	1299	59·6	284	56·2	0·369
	Mean	SD	Mean	SD	Mean	SD	Mean	SD	Mean	SD	Mean	SD	
Average age (years)	46·9	13·0	46·2	12·8	46·9	13·0	47·1	12·8	46·5	13·1	46·5	13·2	0·384
	*n*	%	*n*	%	*n*	%	*n*	%	*n*	%	*n*	%	
Percentage of age groups in sleep duration categories													
18–29 years	914	10·1	46	10·8	206	9·9	350	9·3	252	11·6	60	11·9	0·084
30–39 years	1817	20·3	82	19·3	408	19·7	792	21·0)	434	19·9	101	20·0	0·717
40–49 years	2937	26·8	120	28·3	578	27·9	983	26·1	583	26·8	133	26·3	0·591
50–59 years	2163	24·2	106	25·0	490	23·6	938	24·9	523	23·5	116	23·0	0·670
60–69 years	1365	15·2	60	14·2	319	15·4	596	15·8	313	14·4	77	15·2	0·621
Aged 70 and over	298	3·3	10	2·4	73	3·5	113	3·0	84	3·9	18	3·6	0·315
	Mean	SD	Mean	SD	Mean	SD	Mean	SD	Mean	SD	Mean	SD	
e-GFR (ml/min/1·73 m^2^)	77·8	14·6	77·9	14·8	77·7	14·5	77·8	14·5	78·1	15·2	77·3	14·4	0·830
ASCVD risk													
Waist circumference (cm)	80·6	9·8	82·8	11·8	81·6	10·7	81·6	10·3	81·5	10·3	80·6	9·8	0·042
BMI (kg/m^2^)	23·2.	3·8	23·6	4·3	23·3	3·8	23·2	3·7	23·2	3·8	22·9	3·6	0·076
TC (mg/dl)	201	33	201	33	201	34	201	34	201	33	202	35	0·846
LDL-cholesterol (mg/dl)	117	30	117	30	117	30	117	30	117	30	117	30	0·995
HDL-cholesterol (mg/dl)	62	15	62	15	63	16	62	16	62	16	64	16	0·274
TAG (mg/dl)	97	59	98	58	95	57	98	60	98	59	92	58	0·067
Non-HDL-cholesterol (mg/dl)	139	33	139	33	138	33	139	33	138	33	139	34	0·970
Systolic blood pressure (mmHg)	118	14	118	15	118	14	118	15	118	14	117	14	0·569
Diastolic blood pressure (mmHg)	75	12	75	12	75	12	75	12	75	12	74	11	0·561
Heart rate (bpm)	72	11	73	11	72	11	72	11	72	11	72	11	0·327
FBG (mg/dl)	99	16	100	20	99	16	99	16	100	16	99	17	0·844
HbA1c (%)	5·74	0·55	5·77	0·69	5·73	0·54	5·74	0·54	5·73	0·55	5·73	0·55	0·775
	Median	IQR	Median	IQR	Median	IQR	Median	IQR	Median	IQR	Median	IQR	
* HOMA-IR	1·17	0·82/1·77	1·25	0·90/2·02	1·16	0·82/1·77	1·17	0·83/1·77	1·19	0·82/1·76	1·10	0·75/1·69	0·063
* CRP (mg/dl)	0·03	0·02/0·08	0·03	0·02/0·09	0·03	0·02/0·08	0·03	0·02/0·08	0·04	0·02/0·08	0·04	0·03/0·09	0·205
Lifestyle behaviours													
	Mean	SD	Mean	SD	Mean	SD	Mean	SD	Mean	SD	Mean	SD	
Frequency of fish consumption (d/week)	2·14	1·28	1·94	1·40	2·04	1·26	2·12	1·25	2·26	1·29	2·28	1·35	<0·0001
Amount of fish consumption (g/week)	134	85	121	94	128	84	133	82	142	87	144	93	<0·0001
	*n*	%	*n*	%	*n*	%	*n*	%	*n*	%	*n*	%	
Aerobic exercise habit	2086	23·3	80	18·9	436	21·0	826	21·9	595	27·3	151	29·9	<0·0001
Intensive physical activity	4146	46·3	173	40·8	917	44·2	1743	46·2	1068	49·0	248	49·1	0·002
Cigarette smoking habit	1310	14·6	84	19·8	326	15·7	559	14·8	280	12·8	61	12·1	0·001
	Mean	SD	Mean	SD	Mean	SD	Mean	SD	Mean	SD	Mean	SD	
Alcohol intake habit (g/week)	77	108	75	102	77	109	79	111	76	104	79	105	0·883

ASCVD, atherosclerotic CVD; e-GFR, estimated glomerular filtration rate; TC, total cholesterol; FBG, fasting blood glucose; HOMA-IR, homoeostasis model assessment of insulin resistance; CRP, C-reactive protein.

* IQR.

The frequency of fish intake indicates the average number of days of fish intake per week. Aerobic exercise habit was defined as performing aerobic exercise more than 30 min at least twice per week. Intensive physical activity was defined as walking or engaging in similar physical activity for 1 h or more per d in daily life. The average weekly alcohol intake was calculated from the number of alcoholic drinks consumed per week and the amount of alcohol consumed per drink (ethanol equivalent (g/week))^(17)^.

### Association between fish consumption, sleep duration and leucocytes count

To comprehensively and multilaterally examine the relationship between fish consumption, sleep duration and leucocytes count, we divided the study into the following four sections:

#### Comparing participant characteristics according to sleep duration

Sleep duration increased with increasing amount and frequency of fish consumption (both *P* < 0·0001). The participants engaging in more frequent aerobic exercise habits and those with more significant intensive physical activity had longer sleep duration (*P* < 0·0001 and 0·002). Nevertheless, greater cigarette smoking was associated with shorter sleep duration (*P* = 0·001), and greater waist circumference and HOMA-IR were associated with shorter sleep duration (*P* = 0·043 and 0·016). No relevant associations between other variables and sleep duration were observed.

#### Univariate and multivariate linear regression analysis to determine factors affecting average sleep duration

Next, to examine the relationship between sleep duration and fish consumption in detail, we conducted univariate and multivariate linear regression analyses using sleep duration as the dependent variable and participant characteristics including fish consumption as independent variables.

In all cases, the amount of fish consumption, aerobic exercise habits and intensive daily physical activity were positively associated with sleep duration. Still, waist circumference, HOMA-IR and cigarette smoking were negatively associated with sleep duration. The factors mentioned above were entered into the multivariate linear regression model. Multivariate linear regression analyses revealed that the amount of fish consumption and aerobic exercise habit were independent positive determinants of sleep duration, and cigarette smoking was a negative determinant of sleep duration in both sexes. However, waist circumference and HOMA-IR were negative determinants of sleep duration in males but not in females (Table 2).

**Table 2.** Univariate and multivariate linear regression analysis to distinguish factors affecting sleep duration

All cases (*n* 8954)					Males (*n* 5278)				Females (*n* 3676)			
	Univariate		Multivariate		Univariate		Multivariate		Univariate		Multivariate	
	*r*	*P*	*β*	*P*	*r*	*P*	*β*	*P*	*r*	*P*	*β*	*P*
Male sex	–0·003	0·764										
Age	–0·007	0·507			–0·013	0·353			–0·007	0·664		
e-GFR	0·003	0·779			0·012	0·399			0·007	0·690		
ASCVD risk												
Waist circumference	–0·022	0·037	–0·004	0·774	–0·037	0·007	–0·022	0·258	0·002	0·904		
TC	0·008	0·468			0·011	0·437			0·003	0·841		
LDL-C	0·001	0·959			0·004	0·756			–0·005	0·756		
HDL-C	0·015	0·156			0·021	0·125			0·003	0·836		
TG	0·001	0·947			–0·003	0·822			0·016	0·320		
Non-HDL-C	0·001	0·94			0·002	0·910			0·002	0·909		
Systolic blood pressure	–0·002	0·872			–0·005	0·701			0·008	0·611		
Diastolic blood pressure	–0·003	0·778			–0·002	0·888			–0·002	0·895		
Heart rate	–0·014	0·196			–0·011	0·435			–0·019	0·252		
FBG	–0·005	0·663			–0·014	0·296			–0·006	0·700		
HbA1c	–0·009	0·376			–0·011	0·421			–0·004	0·795		
*HOMA-IR	–0·034	0·005	–0·039	0·011	–0·046	0·003	–0·048	0·013	0·003	0·880		
Lifestyle behaviours												
Amount of fish consumption	0·071	<0·0001	0·076	<0·0001	0·058	<0·0001	0·065	<0·0001	0·094	<0·0001	0·083	<0·0001
Aerobic exercise habit	0·066	<0·0001	0·059	<0·0001	0·064	<0·0001	0·055	0·001	0·075	<0·0001	0·055	0·001
Intensive physical activity	0·041	<0·0001	0·014	0·260	0·037	0·007	0·006	0·686	0·049	0·003	0·027	0·119
Cigarette smoking habit	–0·043	<0·0001	–0·051	<0·0001	–0·041	0·003	–0·045	0·003	–0·047	0·004	0·038	0·021
Alcohol consumption habit	–0·011	0·913			0·010	0·446			–0·002	0·890		
Multiple *R* = 0·124, *F* = 17·435, *P* < 0·0001					Multiple *R* = 0·119, *F* = 9·977, *P* < 0·0001				Multiple *R* = 0·122, *F* = 13·885, *P* < 0·0001			

e-GFR, estimated glomerular filtration rate; ASCVD, atherosclerotic CVD; TC, total cholesterol; FBG, fasting blood glucose; HOMA-IR, homoeostasis model assessment of insulin resistance; CRP, C-reactive protein; *r*, correlation coefficient; *β*, standard partial regression coefficient.

Since the waist circumference and BMI are well known to be highly correlated with each other, waist circumference, which is a better indicator of visceral obesity than the BMI and also serves as an indicator of energy intake, was entered into the univariate linear regression model as an independent variable. *IQR

**Fig. 3.**
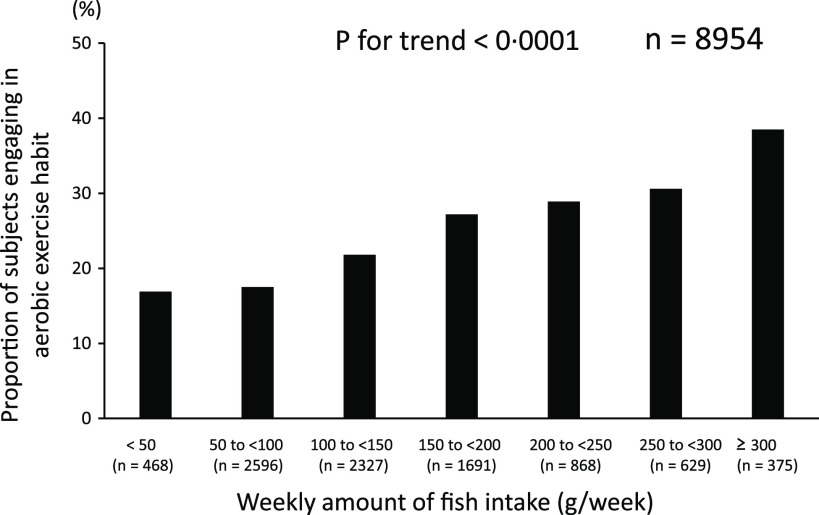
Relationship between fish consumption and aerobic exercise habits.

**Fig. 4.**
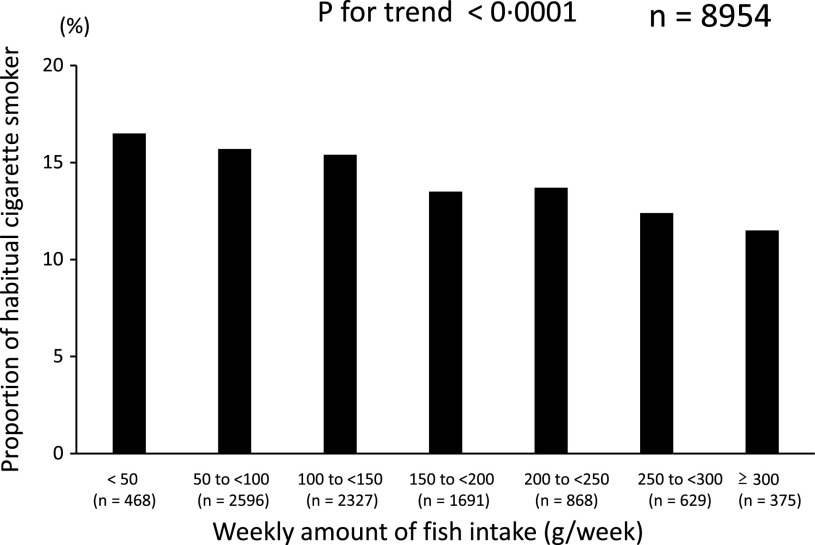
Relationship between fish consumption and cigarette smoking habits.

Next, we examined the relationship between fish consumption and lifestyle behaviours in the subjects of this study to verify various whether eating habits, mainly fish consumption, are related to good lifestyle habits. In fact, as the weekly amount of fish consumption increased, the proportion of subjects engaging in habitual aerobic exercise increased, and the proportion of habitual cigarette smokers decreased (*P* < 0·0001 and 0·001) (Fig. 3 and 4). In two-way ANOVA, we found no interaction between the amount of fish consumption and the lifestyle behaviours mentioned above regarding their relationship with sleep duration (*P*_for interaction_ = 0·458). These analyses suggest that lifestyle behaviours such as aerobic exercise, cigarette smoking and high fish consumption were non-interactive and independent determinants of sleep duration.

#### Impact of association of sleep duration and leucocytes count on fish consumption

**Fig. 5.**
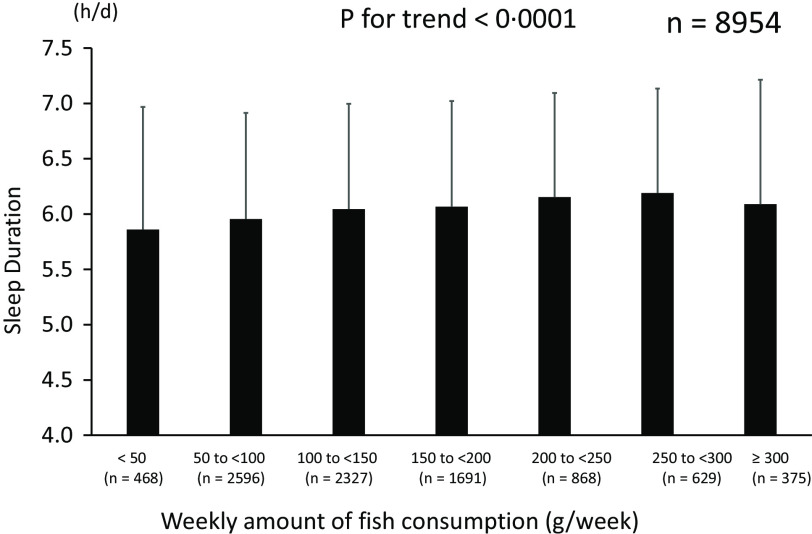
Relationship between fish consumption and sleep duration. Mean values of sleep duration were 5·86 ± 1·11 h, 5·96 ± 0·96 h, 6·05 ± 0·95 h, 6·07 ± 0·95 h, 6·15 ± 0·94 h, 6·19 ± 0·94 h and 6·09 ± 1·12 h in groups with 1, 2, 3, 4, 5, 6 or 7 instances of fish consumption per week, respectively.

**Fig. 6.**
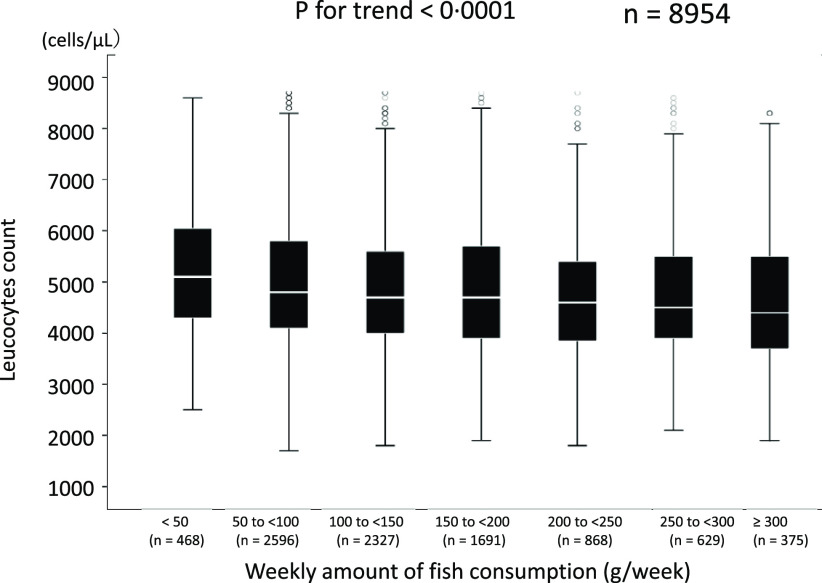
Relationship between fish consumption and leucocytes count. Median (IQR) leucocytes counts were 5100 (4300/6075) cells/μl, 4800 (4100/5800) cells/μl, 4700 (4000/5600) cells/μl, 4700 (3900/5700) cells/μl, 4600 (3825/5400) cells/μl, 4500 (3900/5500) cells/μl and 4400 (3700/5500) cells/μl in groups with 1, 2, 3, 4, 5, 6 or 7 instances of fish consumption per week, respectively.

Next, we examined the relationship between fish consumption as an independent variable and leucocytes count and sleep duration as dependent variables; that is, the sleep duration and leucocytes count of the participants were compared according to the amount of fish consumption as a categorical variable. A higher amount of fish consumption was associated with more sleep duration (Fig. 5) and a lower leucocytes count (Fig. 6). Then, we also performed a multivariate linear regression analysis using leucocytes count as the dependent variable and fish consumption and participant characteristics as independent variables. As a result, the amount of fish consumption was an independent negative determinant of leucocytes count (all cases: *β* = –0·091, *P* < 0·0001, males: *β* = –0·104, *P* < 0·0001, and females: *β* = –0·070, *P* < 0·0001) (online Supplementary Table 3).

#### Relationship between sleep duration and leucocytes count

**Fig. 7.**
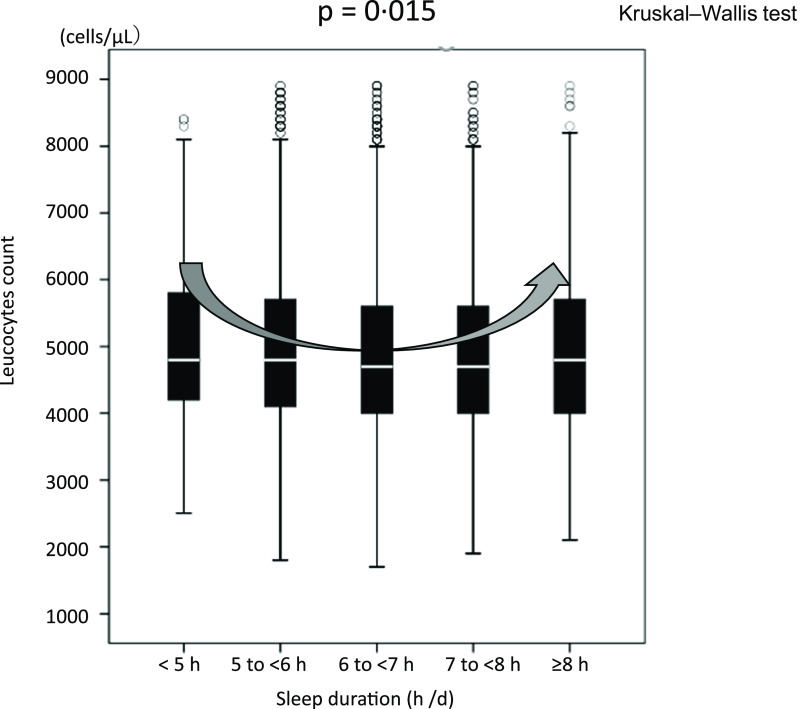
Relationship between sleep duration and leucocytes count. Median (IQR) leucocytes counts according to sleep duration category: 4800 (4200/5875) cells/μl, 4800 (4100/5700) cells/μl, 4700 (4000/5600) cells/μl, 4700 (4000/5600) cells/μl and 4800 (4000/5700) cells/μl. The leucocytes counts may be higher in both short and long sleep durations than adequate sleep duration. IQR, interquartile range.

Fig. 7 shows a significant U-shaped association between sleep duration and leucocytes count, with 6–7 h of sleep as the lower value (*P* = 0·015). The leucocytes count increased as sleep duration decreased, and the leucocytes count was the lowest value when the sleep duration was 6–7 h. The leucocytes count increased again after >7 h of sleep. Further, to assess whether higher fish consumption leads to longer sleep duration, two-way ANOVA was performed with leucocytes count as the dependent variable and amount of fish consumption and sleep duration as independent variables. The results showed that high fish consumption and long sleep duration were non-interactive and independent determinants of leucocytes count (*P*_for interaction_ = 0·966). Therefore, a higher amount of fish consumption is not associated with longer sleep duration, that is, amount of fish consumption and sleep duration are independently associated with leucocytes count.

From the above results, we showed the overall relation between fish consumption, sleep duration and leucocytes count.

## Discussion

This study yielded the following results: inappropriate sleep duration (i.e. short/long sleep duration) may be a symptom of chronic activation of the systemic inflammatory response. However, high fish consumption may be associated with a low leucocytes count and increased sleep duration. Moreover, we found a significant U-shaped association between leucocytes count and sleep duration, with 6–7 h of sleep as the low value. Therefore, not only short sleep duration but also long sleep duration may be associated with increased inflammation. The results suggest that higher fish consumption may be related to a lower leucocytes count in the existence of adequate sleep duration and healthy lifestyle behaviours.

This study showed multiple associations between fish consumption, sleep duration and leucocytes count. Consequently, habitual short sleep duration with reduced fish consumption additively induces chronic systemic inflammation. Moreover, the enhancement of chronic inflammation via long sleep duration may surpass the anti-inflammatory effect of high fish consumption. These associations may partially explain the increased incidence of ASCVD, not only in people with short sleep duration but also in those with long sleep duration^(24,25)^.

Notably, the effects on sleep quality are also supported by plausible physiological mechanisms from animal models that show that *n*-3 PUFA promote serotonin secretion^(26)^, which is a source of melatonin that regulates sleep^(27)^, in turn supporting an optimal circadian clock rhythm. Oily fish is the main contributor of dietary vitamin D. Expression of vitamin D receptors is high in several sleep-involved nuclei in the hypothalamus and brainstem. Vitamin D has been suggested to be inversely associated with sleep disorders^(28)^. A population-based study demonstrated that consumption of oily fish was associated with improved sleep quality. Even for people who consume more than the recommended amount of fish, increased fish consumption is associated with more improvement in sleep quality^(29)^. Based on these reports, fish consumption significantly improves sleep quality, leading to adequate sleep duration.

The ATTICA study, with a cross-sectional design involving the citizens of Athens, Greece (a Mediterranean country similar to Japan in terms of fish consumption (i.e. a traditional diet with high fish intake)), showed a negative correlation between the amount of fish consumption and several inflammatory biomarkers, including the leucocytes count^(30)^. This finding supports our results. The outcomes of this study may represent common phenomena observed in populations with high fish consumption.

Short or long sleep duration induces the activation of the autonomic nerve system, secretion of elevated levels of catecholamines and inflammation^(31–33)^. Thus, inadequate sleep duration is associated with increased leucocytes count^(6,34,35)^, leading to ASCVD. Conversely, as demonstrated in Fig. 7, it is easy to understand that the leucocytes count is lower in those with 6 to <7 h of sleep, usually referred to as adequate sleep, than in those with inappropriately short and long periods of sleep. Alternatively, long sleep duration, involved in metabolic disorders such as the prevalence of metabolic syndrome, may also increase the inflammatory response^(36,37)^.

Detailed results could have been obtained if serum levels of *n*-3 PUFA were measured. It has been reported that the correlation coefficient for the association between the frequency of fish consumption, as estimated by the FFQ, and fatty acid concentration in the erythrocyte membrane is 0·42–0·51^(38)^. Therefore, we speculate that the index of the amount of fish consumption calculated from the frequency of fish intake used in this study was a true reflection of fish consumption by the study participants. Furthermore, we recently reported that the frequency and amount of fish consumption were positively correlated with *n*-3 PUFA consumption^(16)^. These results suggest that fish consumption is related positively to the amount of bioactive *n*-3 PUFA absorbed by the body organs.

Finally, we propose that high fish consumption may be associated with adequate sleep duration, and that it suppresses inflammation associated with inappropriately short and long sleep durations. Prospective cohort studies examining the relationship between sleep duration and inflammatory responses modified by fish consumption are needed to test this concept.

### Study limitations

First, the types and amounts of fish included in the diet, especially the consumption of oily fish, which is known to be high in PUFA content, were not considered in the analysis. Furthermore, there was no information in this study on the consumption of other foods, such as meat, vegetables and fruits, that can potentially affect the serum lipid levels, anti-inflammation and antioxidants. Second, self-reporting questionnaire, especially reporting the presence of intensive physical activity as in this study, depends on the subjectivity of the study participants. Therefore, the self-reporting questionnaire assessment may lack objectivity; for example, it does not quantify physical activity. Thus, we should use a detailed internationally standardised questionnaire^(39,40)^ or standard variables to facilitate international comparisons (i.e. lifestyle, dietary habits and physical activity). We should also evaluate sleep quality using the internationally standardised Pittsburgh Sleep Quality Index^(41)^. Third, most study participants were White people who worked in the Tokyo metropolitan area^(42)^. In addition, older people refrained from health checkups due to concerns about the COVID-19 pandemic after January 2019. Therefore, the results of this study cannot be generalised to other different populations. Fourth, intake of sleeping pills, which affects sleep hours and quality, has not been assessed. Finally, in this study, we could not define the sleep duration of long sleep duration, which is a condition that enhances inflammatory response. The group with a sleep duration of 8 h was composed of a small number of participants and was therefore not further subdivided into 8 h≥ group. That is, there may be a difference in the influence on leucocytes count between 8 h and 12 h (most prolonged sleep duration in this study participants) of sleep.

### Clinical implications

This study excluded leucocytes counts greater than 9000 cells/μl to exclude infectious diseases. Therefore, we may have excluded high-risk patients with a leucocytes count > 9000 cells/μl without infectious disease. However, in our study design, even though we restricted the study population to a normal leucocytes count range, a higher leucocytes count was still observed in inadequate short and excessive sleep duration, which may have helped stratify the risk of ASCVD.

### Conclusions

The results imply that higher fish consumption may be associated with a lower leucocytes count in the presence of adequate sleep duration and healthy lifestyle behaviours. That is, not only short sleep duration but also long sleep duration was associated with a higher leucocytes count. These associations may partially explain the preventive effects of higher fish consumption on ASCVD events. Further studies are required to clarify the causal relationships in these results.

## Supplementary material

For supplementary material accompanying this paper visit https://doi.org/10.1017/S0007114522002896. click here to view supplementary material
