# Supplementary material for: Inadequate sleep duration may attenuate the anti-inflammatory effects of fish consumption in a healthy Japanese population: a cross-sectional study
Source: Br J Nutr. 2022 Oct 19;129(12):2142–52. doi: 10.1017/S0007114522002896 (PMC10197088; doi:10.1017/S0007114522002896)
Supplement: Supplementary file 1 [file S0007114522002896sup.zip › S0007114522002896sup001.pdf]

Supplemental Table 2

Trends in Mean Fish and Shellfish Intakes
by Food Groups, 2001-2019 (by Gender and Age)

		2001			2002			2003			2004			2005			2006			2007			2008		
		n	Mean	SD	n	Mean	SD	n	Mean	SD	n	Mean	SD	n	Mean	SD	n	Mean	SD	n	Mean	SD	n	Mean	SD
ALL:	Total	12,481	94.0	86.4	11,491	88.2	78.1	11,105	86.7	80.1	8,762	82.6	76.8	8,895	84.0	77.5	9,423	80.2	76.6	8,885	80.2	76.7	9,129	78.5	73.6
	1–6	797	38.5	38.3	687	40.7	43.8	639	38.1	39.7	520	34.5	33.1	463	39.5	43.3	559	35.8	43.5	488	35.9	40.2	421	30.8	36.5
	7–14	1,171	65.0	60.5	916	59.2	52.9	939	60.6	55.8	770	57.7	56.8	741	57.8	52.4	815	59.1	57.2	795	53.1	52.2	704	53.8	52.8
	15–19	688	80.3	73.5	615	73.0	65.4	563	72.2	74.4	435	65.8	68.5	429	68.1	65.6	458	68.6	75.3	393	60.9	58.2	360	62.0	64.3
	20–29	1,282	80.7	80.1	1,148	73.3	70.8	1,068	71.5	72.7	803	67.1	68.8	803	69.8	73.6	780	64.1	69.0	665	69.6	71.6	703	63.6	70.1
	30–39	1,545	82.1	81.8	1,380	77.3	70.9	1,393	73.3	75.7	1,124	71.5	71.8	1,053	72.4	74.5	1,254	70.2	73.4	1,201	65.1	67.9	1,044	62.7	66.1
	40–49	1,666	99.4	87.0	1,420	91.8	81.1	1,331	93.2	82.1	1,045	82.5	78.0	1,090	80.7	77.4	1,101	76.1	68.7	1,107	74.2	73.9	1,037	72.7	72.8
	50–59	1,963	120.8	93.3	1,887	108.2	87.0	1,730	105.5	87.2	1,374	104.9	87.1	1,346	99.7	84.0	1,483	95.2	83.4	1,268	98.6	81.1	1,368	91.5	77.9
	60–69	1,716	123.0	101.1	1,686	114.3	87.9	1,642	110.9	88.8	1,368	107.2	83.6	1,426	108.1	86.9	1,341	105.2	89.1	1,426	104.6	84.8	1,615	103.0	80.6
	70 and over	1,653	101.0	82.6	1,752	96.1	73.0	1,800	96.5	78.2	1,323	92.0	72.4	1,544	96.0	74.4	1,632	93.2	74.4	1,542	96.1	83.5	1,877	88.4	72.1
	(Regrouped)																								
	20 and over	9,825	102.9	90.1	9,273	95.6	80.8	8,964	93.8	82.8	7,037	89.9	79.5	7,262	90.5	80.4	7,591	86.4	78.8	7,209	87.2	79.9	7,644	84.1	75.6
	75 and over	–	–	–	1,018	94.4	72.4	1,062	91.6	76.5	797	91.5	73.7	910	92.6	68.9	1,008	91.8	73.6	954	91.2	83.2	1,168	83.9	68.7
Males:	Total	5,852	103.2	93.3	5,377	97.9	84.8	5,263	95.3	88.5	4,135	89.9	83.4	4,163	92.0	83.8	4,443	87.6	83.8	4,164	88.8	84.2	4,269	87.9	80.9
	1–6	396	38.0	39.3	346	40.1	45.6	347	40.5	42.6	263	36.2	33.2	229	41.6	47.2	276	35.2	43.2	243	38.4	43.1	204	30.7	36.7
	7–14	591	69.3	63.9	452	62.3	56.2	472	60.1	55.7	419	59.4	59.8	365	60.5	53.2	422	58.0	57.9	392	56.0	51.8	367	56.3	52.7
	15–19	358	81.3	76.0	301	79.7	69.9	272	82.5	87.0	239	63.0	66.6	218	72.1	65.8	239	73.9	82.0	201	64.7	60.6	190	66.2	72.9
	20–29	587	89.2	84.2	540	80.4	75.8	516	75.2	79.7	353	71.7	74.0	399	73.5	78.4	367	66.0	75.1	304	76.8	77.4	337	74.0	73.8
	30–39	702	93.1	93.2	657	86.3	76.1	671	83.7	82.5	525	79.3	76.0	508	81.0	80.4	583	78.9	78.9	540	73.7	76.2	476	73.1	74.3
	40–49	798	113.4	93.9	677	104.5	85.9	643	104.6	92.5	480	94.7	87.5	500	93.8	85.2	519	86.3	73.7	537	84.8	83.1	498	84.7	82.7
	50–59	930	133.0	99.7	891	121.7	95.3	816	118.6	97.0	649	116.2	95.8	623	112.0	90.9	702	106.1	93.0	587	109.9	89.7	614	101.9	83.8
	60–69	804	136.6	106.8	801	130.4	95.8	771	124.3	98.3	631	121.4	90.2	648	120.6	96.2	618	118.0	97.6	664	116.1	90.5	755	116.7	89.8
	70 and over	686	111.9	90.6	712	107.7	77.3	755	108.9	84.9	576	100.7	79.0	673	104.9	78.5	717	104.7	82.2	696	108.1	94.2	828	100.4	79.6
	(Regrouped)																								
	20 and over	4,507	115.0	97.3	4,278	107.6	87.6	4,172	104.7	91.7	3,214	100.3	86.9	3,351	100.2	87.2	3,506	96.3	86.5	3,328	97.8	88.2	3,508	95.7	83.3
	75 and over	–	–	–	388	107.3	75.7	410	103.0	80.8	332	100.8	81.1	355	102.0	71.3	414	106.4	80.7	410	103.4	94.4	487	96.0	78.1
Females:	Total	6,629	84.1	75.4	6,114	79.7	70.5	5,842	78.9	70.8	4,627	76.1	69.8	4,732	76.9	70.9	4,980	73.6	68.8	4,721	72.6	68.6	4,860	70.2	65.3
	1–6	401	38.6	37.1	341	41.4	41.9	292	35.2	35.7	257	32.7	33.0	234	37.3	39.2	283	36.4	43.8	245	33.4	36.9	217	31.0	36.3
	7–14	580	59.7	54.8	464	56.3	49.3	467	61.2	55.9	351	55.7	53.0	376	55.1	51.5	393	60.4	56.6	403	50.4	52.5	337	51.0	52.9
	15–19	330	75.8	65.8	314	66.5	60.2	291	62.7	59.0	196	69.2	70.9	211	64.0	65.4	219	62.7	67.0	192	56.9	55.4	170	57.4	53.0
	20–29	695	71.3	72.4	608	67.1	65.5	552	68.0	65.3	450	63.5	64.2	404	66.3	68.4	413	62.3	63.1	361	63.6	65.7	366	54.1	65.3
	30–39	843	72.4	68.5	723	69.1	64.9	722	63.7	67.4	599	64.6	67.4	545	64.4	67.7	671	62.7	67.3	661	58.1	59.4	568	54.1	57.0
	40–49	868	83.9	73.0	743	80.2	74.6	688	82.5	69.4	565	72.2	67.3	590	69.6	68.3	582	67.0	62.6	570	64.2	62.5	539	61.6	60.3
	50–59	1,033	106.9	81.5	996	96.2	76.8	914	93.8	75.5	725	94.8	77.1	723	89.2	76.0	781	85.4	72.3	681	88.9	71.6	754	83.1	71.7
	60–69	912	108.6	87.1	885	99.8	77.4	871	99.1	77.7	737	95.1	75.4	778	97.8	76.8	723	94.2	79.7	762	94.5	78.2	860	90.9	69.4
	70 and over	967	92.7	74.9	1,040	88.1	68.8	1,045	87.6	71.7	747	85.3	66.2	871	89.1	70.3	915	84.1	66.3	846	86.3	72.0	1,049	78.9	64.1
	(Regrouped)																								
	20 and over	5,318	90.7	78.2	4,995	85.3	73.0	4,792	84.3	72.9	3,823	81.2	71.5	3,911	82.1	73.0	4,085	78.0	70.4	3,881	78.2	70.7	4,136	74.3	66.8
	75 and over	–	–	–	630	86.5	69.2	652	84.4	72.8	465	84.8	67.2	555	86.6	66.6	594	81.7	66.4	544	82.0	72.3	681	75.3	59.7

Supplemental T Supplemental Table 2

Trends in Mean Fis Trends in Mean Fish and Shellfish Intakes

by Food Groups, 2 by Food Groups, 2001-2019 (by Gender and Age)

		2009			2010			2011			2012			2013			2014			2015			2016		
		n	Mean	SD	n	Mean	SD	n	Mean	SD	n	Mean	SD	n	Mean	SD	n	Mean	SD	n	Mean	SD	n	Mean	SD
ALL:	Total	9,006	74.2	74.0	8,815	72.5	70.9	8,247	72.7	71.8	32,228	70.0	71.3	7,801	72.8	71.2	8,047	69.4	68.5	7,456	69.0	68.8	26,133	65.6	63.1
	1-6	467	32.1	36.0	461	28.8	34.4	394	32.8	36.3	1,645	30.8	35.8	374	26.4	29.7	345	26.2	31.3	353	30.5	33.4	1,244	29.5	32.1
	7-14	759	54.4	53.6	739	53.7	57.4	720	50.7	53.7	2,556	49.8	51.6	609	48.3	53.1	620	47.1	48.4	597	45.8	46.6	1,988	46.1	45.3
	15-19	403	59.5	64.8	386	54.1	61.6	380	50.3	50.7	1,301	52.3	58.3	337	52.6	58.7	355	52.7	58.2	334	55.6	56.7	1,050	47.0	51.6
	20-29	659	57.2	65.4	649	60.0	61.8	587	57.2	66.7	2,255	55.3	64.3	557	55.6	60.1	491	54.9	61.7	470	52.9	58.8	1,489	50.4	57.8
	30-39	1,079	61.9	71.2	1,083	59.2	64.8	1,004	60.9	66.2	3,650	59.4	65.2	788	59.3	64.5	797	55.7	60.8	709	58.5	66.0	2,557	53.2	58.4
	40-49	1,135	67.0	70.6	1,077	65.7	69.4	1,038	61.8	66.0	3,948	60.4	66.8	989	60.5	64.2	1,009	54.3	59.0	1,035	54.0	62.6	3,400	55.7	64.6
	50-59	1,254	86.0	75.1	1,168	87.2	76.9	1,076	81.6	75.0	4,110	77.1	75.1	961	81.2	74.5	1,027	75.0	72.2	959	75.1	73.2	3,263	67.2	63.8
	60-69	1,505	99.4	87.8	1,558	95.8	78.4	1,349	97.7	81.0	5,851	93.4	81.3	1,408	94.6	78.3	1,548	89.0	75.2	1,373	86.4	74.3	4,948	84.6	65.7
	70 and over	1,745	86.2	72.8	1,694	83.0	70.0	1,699	89.6	75.1	6,912	86.5	73.8	1,778	91.1	74.9	1,855	86.4	72.3	1,626	89.1	73.0	6,194	83.0	65.9
	(Regrouped)																								
	20 and over	7,377	79.8	76.7	7,229	78.2	72.8	6,753	78.6	74.6	26,726	75.6	74.0	6,481	78.8	73.3	6,727	74.5	70.6	6,172	74.2	71.4	21,851	70.5	65.2
	75 and over	1,132	84.9	72.5	1,071	80.8	69.2	1,090	84.5	69.3	4,365	84.0	72.3	1,088	88.0	70.9	1,154	85.8	73.7	1,051	85.3	70.2	4,066	81.9	64.6
Males:	Total	4,180	82.3	81.8	4,153	80.2	77.6	3,839	80.9	79.0	15,084	77.4	78.4	3,684	79.9	77.2	3,786	77.1	76.0	3,502	74.7	74.2	12,202	71.8	68.5
	1-6	238	32.4	38.8	236	31.3	36.1	209	33.9	36.2	846	31.5	37.1	197	26.4	29.6	181	28.9	33.5	182	31.8	33.8	611	31.7	33.4
	7-14	377	56.8	55.7	390	58.4	64.8	352	52.2	55.6	1,271	53.6	55.0	314	50.7	58.0	320	46.0	50.7	315	46.6	45.9	1,045	48.5	47.6
	15-19	206	62.2	73.7	193	59.9	67.3	193	56.2	54.4	702	54.9	60.6	175	61.6	62.7	173	54.3	60.8	165	56.0	58.5	559	51.5	50.3
	20-29	297	57.5	70.3	310	63.9	66.1	259	66.6	72.1	1,078	59.9	71.2	273	58.6	62.0	219	57.3	67.2	225	52.5	55.9	710	51.9	62.0
	30-39	500	73.5	81.3	507	66.9	72.5	479	68.7	74.2	1,715	66.5	71.1	368	67.5	70.1	376	62.8	65.9	347	66.5	74.1	1,207	60.9	64.6
	40-49	539	77.8	78.1	517	72.5	71.0	480	70.5	71.5	1,835	68.6	72.9	456	67.7	71.4	461	61.0	64.4	453	60.6	70.5	1,581	64.7	71.6
	50-59	603	96.6	80.2	541	99.3	85.5	521	93.1	82.8	1,859	87.5	84.6	455	90.9	84.0	476	87.9	80.7	440	82.6	77.6	1,486	72.5	70.2
	60-69	690	111.7	96.7	726	107.4	84.7	600	109.1	90.0	2,763	105.7	91.3	638	105.0	82.1	741	99.2	85.0	640	95.4	80.9	2,307	93.3	73.8
	70 and over	730	97.4	82.1	733	93.5	78.5	746	102.5	84.0	3,015	96.2	79.6	808	101.9	81.8	839	98.7	79.9	735	98.2	79.3	2,696	91.9	70.2
	(Regrouped)																								
	20 and over	3,359	90.0	84.9	3,334	87.4	79.7	3,085	89.0	82.5	12,265	84.8	81.8	2,998	87.5	79.6	3,112	84.3	78.7	2,840	81.7	77.6	9,987	78.0	71.4
	75 and over	446	97.5	82.7	439	92.0	76.7	469	97.4	79.9	1,828	96.9	78.9	484	99.5	76.6	520	98.7	81.4	457	93.9	76.3	1,711	91.6	70.4
Females:	Total	4,826	67.2	65.7	4,662	65.7	63.6	4,408	65.5	64.1	17,144	63.5	63.6	4,117	66.4	64.7	4,261	62.5	60.2	3,954	64.0	63.2	13,931	60.3	57.4
	1-6	229	31.9	32.8	225	26.2	32.4	185	31.4	36.4	799	30.1	34.3	177	26.3	30.0	164	23.3	28.5	171	29.1	32.9	633	27.4	30.7
	7-14	382	51.9	51.5	349	48.3	47.3	368	49.3	52.0	1,285	46.1	47.8	295	45.8	47.3	300	48.3	45.9	282	45.0	47.4	943	43.4	42.5
	15-19	197	56.8	54.0	193	48.3	54.9	187	44.1	45.8	599	49.2	55.3	162	42.9	52.4	182	51.3	55.8	169	55.3	55.0	491	42.6	52.8
	20-29	362	57.0	61.1	339	56.4	57.5	328	49.8	61.3	1,177	51.2	57.3	284	52.7	58.3	272	53.1	56.9	245	53.3	61.5	779	49.0	53.8
	30-39	579	51.9	59.5	576	52.4	56.4	525	53.7	57.2	1,935	53.1	58.7	420	52.1	58.3	421	49.3	55.1	362	50.9	56.3	1,350	46.3	51.5
	40-49	596	57.3	61.5	560	59.3	67.4	558	54.3	59.9	2,113	53.4	60.1	533	54.2	56.7	548	48.6	53.4	582	48.9	55.3	1,819	48.3	56.9
	50-59	651	76.1	68.6	627	76.8	67.0	555	70.7	65.1	2,251	68.8	65.2	506	72.5	63.5	551	63.9	61.9	519	68.8	68.7	1,777	62.8	57.6
	60-69	815	89.0	78.1	832	85.6	71.0	749	88.7	71.7	3,088	82.2	69.2	770	86.0	73.9	807	79.6	63.5	733	78.6	67.0	2,641	76.9	56.8
	70 and over	1,015	78.1	64.1	961	75.1	61.5	953	79.5	65.7	3,897	78.6	67.7	970	82.1	67.3	1,016	76.2	63.5	891	81.7	66.4	3,498	76.2	61.7
	(Regrouped)																								
	20 and over	4,018	71.2	68.0	3,895	70.4	65.4	3,668	69.9	66.0	14,461	67.7	65.6	3,483	71.3	66.5	3,615	66.0	61.7	3,332	67.8	65.1	11,864	64.3	58.9
	75 and over	686	76.7	63.8	632	73.0	62.3	621	74.8	58.3	2,537	74.3	65.2	604	78.7	64.6	634	75.3	64.8	594	78.8	64.5	2,355	74.8	59.2

Supplemental T Supplemental Table 2

Trends in Mean Fis Trends in Mean Fish and Shellfish Intakes

by Food Groups, 2 by Food Groups, 2001-2019 (by Gender and Age)

(g)

		2017			2018			2019		
		n	Mean	SD	n	Mean	SD	n	Mean	SD
ALL:	Total	6,962	64.4	66.6	6,926	65.1	66.8	5,865	64.1	68.9
	1-6	373	30.4	34.0	389	29.9	36.9	235	29.7	37.3
	7-14	512	46.2	45.8	517	43.8	48.0	454	45.2	48.3
	15-19	283	50.6	56.4	277	49.3	60.7	249	43.3	50.3
	20-29	418	49.5	57.1	428	46.2	55.2	365	50.8	57.9
	30-39	639	51.1	58.3	668	55.7	64.5	460	50.8	61.7
	40-49	978	53.3	60.7	915	53.0	59.7	742	52.8	64.8
	50-59	864	68.8	71.4	908	67.6	68.4	775	59.2	63.4
	60-69	1,186	79.7	73.1	1,174	85.4	76.4	1,046	77.7	74.3
	70 and over	1,709	81.5	72.1	1,650	82.3	67.9	1,539	84.0	77.5
	(Regrouped)									
	20 and over	5,794	68.8	69.2	5,743	70.1	68.8	4,927	68.5	71.5
	75 and over	1,071	78.0	71.9	1,047	81.7	67.2	952	82.9	78.9
Males:	Total	3,319	69.8	72.4	3,260	70.7	72.6	2,782	70.4	76.7
	1-6	197	30.6	33.8	181	33.0	40.0	105	33.5	44.1
	7-14	267	43.9	43.4	273	46.1	52.1	250	46.1	52.5
	15-19	141	63.7	64.6	143	57.8	64.9	130	42.4	53.0
	20-29	219	50.7	59.9	211	49.6	58.6	183	60.0	63.8
	30-39	322	55.8	64.5	314	60.4	67.2	210	56.2	67.8
	40-49	461	59.0	65.9	445	62.4	67.5	351	59.9	72.2
	50-59	392	81.6	83.9	417	70.6	74.1	350	67.4	72.3
	60-69	566	86.2	79.2	548	95.6	86.2	502	85.6	82.3
	70 and over	754	90.1	76.9	728	88.9	72.9	701	92.7	88.0
	(Regrouped)									
	20 and over	2,714	75.5	75.6	2,663	76.5	75.1	2,297	76.3	79.9
	75 and over	476	83.8	74.2	460	89.0	71.6	421	93.1	91.6
Females:	Total	3,643	59.4	60.5	3,666	60.0	60.7	3,083	58.4	60.3
	1-6	176	30.1	34.3	208	27.2	33.8	130	26.6	30.4
	7-14	245	48.7	48.2	244	41.1	42.9	204	44.2	42.7
	15-19	142	37.6	43.3	134	40.2	54.7	119	44.3	47.3
	20-29	199	48.2	54.1	217	43.0	51.5	182	41.6	49.9
	30-39	317	46.3	51.0	354	51.4	61.8	250	46.3	55.9
	40-49	517	48.2	55.2	470	44.0	49.6	391	46.5	56.7
	50-59	472	58.3	56.9	491	65.0	63.2	425	52.5	54.0
	60-69	620	73.8	66.5	626	76.5	65.4	544	70.4	65.4
	70 and over	955	74.7	67.3	922	77.0	63.3	838	76.7	66.6
	(Regrouped)									
	20 and over	3,080	62.9	62.5	3,080	64.6	62.4	2,630	61.7	62.4
	75 and over	595	73.4	69.7	587	76.0	63.0	531	74.7	66.1

注）平成24年、28年の平均値、標準偏差は抽出率等を考慮した全国補正値である。

注）平成24年、28年の平

注）平成24年、28年の平
