# Supplementary material for: Inadequate sleep duration may attenuate the anti-inflammatory effects of fish consumption in a healthy Japanese population: a cross-sectional study
Source: Br J Nutr. 2022 Oct 19;129(12):2142–52. doi: 10.1017/S0007114522002896 (PMC10197088; doi:10.1017/S0007114522002896)
Supplement: Supplementary file 1 [file S0007114522002896sup.zip › S0007114522002896sup002.pdf]

Supplemental Table 3. Univariate and Multivariate Linear Regression Analysis to Distinguish Factors Affecting WBC count

	All cases (n = 8954)				Males (n = 5278)				Females (n = 3676)			
	Univariate		Multivariate		Univariate		Multivariate		Univariate		Multivariate	
	r	p-value	β	p-value	r	p-value	β	p-value	r	p-value	β	p-value
Male sex	0.018	0.096										
Age	0.150	0.167			0.015	0.265			0.007	0.653		
e-GFR	0.008	0.445			0.001	0.920			0.018	0.284		
ASCVD risk												
Waist circumference	-0.008	0.539			-0.023	0.092			-0.006	0.729		
TC	0.012	0.257			0.004	0.767			0.023	0.156		
LDL-C	0.003	0.788			-0.002	0.896			0.004	0.792		
HDL-C	0.015	0.148			0.011	0.442			0.042	0.011	0.044	0.007
TG	-0.004	0.720			-0.008	0.542			-0.013	0.425		
non-HDL-C	0.005	0.654			-0.005	0.971			0.005	0.759		
Systolic blood pressure	0.011	0.312			0.004	0.756			0.006	0.739		
Diastolic blood pressure	0.010	0.330			0.002	0.876			0.008	0.620		
Heart rate	0.002	0.854			0.005	0.715			-0.001	0.944		
FBG	0.005	0.669			-0.002	0.861			0.005	0.744		
HbA1c	0.004	0.707			0.0002	0.990			0.006	0.702		
*HOMA-IR	-0.037	0.002	-0.027	0.025	-0.052	< 0.0001	-0.041	0.008	-0.025	0.211		
Lifestyle behaviors												
Amount of fish consumption	-0.095	< 0.0001	-0.091	< 0.0001	-0.103	< 0.0001	-0.104	< 0.0001	-0.087	< 0.0001	-0.070	< 0.0001
Aerobic exercise habit	-0.078	< 0.0001	-0.065	< 0.0001	-0.091	< 0.0001	-0.074	< 0.0001	-0.059	< 0.0001	-0.047	0.004
Intensive physical activity	-0.030	0.004	0.006	0.615	-0.021	0.128	0.013	0.415	-0.044	0.007	-0.018	0.295
Cigarette smoking habit	0.155	< 0.0001	0.143	< 0.0001	0.135	< 0.0001	0.131	< 0.0001	0.183	< 0.0001	0.174	< 0.0001
Alcohol consumption habit	-0.011	0.282			-0.014	0.322			-0.009	0.585		
	Multiple R = 0.192, F = 51.007, p < 0.0001				Multiple R = 0.196, F = 33.721, p < 0.0001				Multiple R = 0.207, F = 32.702, p < 0.0001			

Abbreviations are as in Table 1. r = correlation coefficient; β = standard partial regression coefficient

Since the waist circumference and body mass index are well known to be highly correlated with each other, waist circumference, which is a better indicator of visceral obesity than the body mass index and also serves as an indicator of energy intake, was entered into the univariate linear regression model as an independent variable.
