# Supplementary material for: Inadequate sleep duration may attenuate the anti-inflammatory effects of fish consumption in a healthy Japanese population: a cross-sectional study
Source: Br J Nutr. 2022 Oct 19;129(12):2142–52. doi: 10.1017/S0007114522002896 (PMC10197088; doi:10.1017/S0007114522002896)
Supplement: Supplementary file 1 [file S0007114522002896sup.zip › S0007114522002896sup003.pdf]

Supplemental Table 1. Questionnaire

How to Fill in the OCR

= Advice to All Who Are Going to Receive the Health Checkup =

= Please follow the instructions given below! =

- Enter your responses using a **black pencil** or **mechanical pencil** (**ball-point pen is unacceptable**); see the examples below.
- This OCR sheet will be mechanically read. Do not bend it or expose it to water or dirt.

[Examples of entering numerals]

™ ◎ Good entry: 1 2 3 4 5 6 7 8 9 0

× Bad entry

- 1 — Adding a hook
- 2 — Adding a loop
- 4 — May be mistaken read as "9" ⇒ 4 — Do not join the two upper vertical lines while extending the horizontal line to the right
- 6 — Uncertain as to which of "0" or "6" is meant
- 9 — Exceeding the space for entry

[Examples of entering marks]

™ ◎ Good entry: ✓ or ○

× Bad entry

- Encircling the frame
- Exceeding the space for entry
- ✓ — Too small

[Other points requiring care]

Age : 0 4 (month) 0 3 (day) ⇒ Add "0" before one-digit number

[Please ensure that you have answered all the questions!]

If your responses to the questionnaire are incomplete, you will have to sit for an inquiry on the date of the health checkup.

In such cases, the time taken for the checkup will be longer than usual. Please understand it.

Name: years old I D | | | | | | | | | | | 1 1

Questionnaire

1. Are you currently suffering from any illness for which you are now receiving treatment? Please check the appropriate boxes.

- Please enter the name of illness on which you are receiving doctor's care through periodical visit to a medical facility.
- If you have no illness for which you are receiving outpatient care, please check the column, "No illness warranting outpatient care."

☐ No illness warranting outpatient care

☐ Taking medication for hypertension (since years ago) (Using blood pressure-lowering medication)
☐ Have hypertension, but do not take any medication (Receiving doctor's care through periodic visits to a medical facility)

☐ Taking medication for diabetes mellitus (since years ago) (Using insulin injections or blood glucose-lowering medication)
☐ Have diabetes mellitus, but do not take any medication (Receiving doctor's care through periodic visits to a medical facility)

☐ Taking medication for dyslipidemia (hyperlipidemia) (since years ago) (Using cholesterol- or triglyceride-lowering medication)
☐ Have dyslipidemia (hyperlipidemia) but do not take any medication (Receiving doctor's care through periodic visits to a medical facility)

☐ Diagnosed by a doctor as having heart disease (angina pectoris, myocardial infarction, etc.) or receiving treatment for heart disease (**Diagnosis:**)

☐ Diagnosed by a doctor as having stroke (cerebral hemorrhage, cerebral infarction, etc.) or receiving treatment for stroke (**Diagnosis:**)

☐ Diagnosed by a doctor as having chronic kidney disease or renal failure, or receiving treatment (hemodialysis, etc.) for chronic kidney disease/renal failure

☐ Taking medication for anemia ☐ Have anemia, but do not take any medication (Receiving doctor's care through periodic visits to a medical facility)

☐ Taking medication for gout (hyperuricemia)

☐ Liver disease Diagnosis ( )	☐ Gastroduodenal disease Diagnosis ( )
☐ Colorectal disease Diagnosis ( )	☐ Gallstone
☐ Urogenital disease Diagnosis ( )	☐ Orthopedic disease Diagnosis ( )
☐ Respiratory system disease Diagnosis ( )	☐ Neuropsychiatric disease Diagnosis ( )
☐ Others Diagnosis ( )	

2. Inquiry before hearing test and ophthalmological test. Please check the appropriate boxes.

[Hearing test]	[Ophthalmological test]
☐ Suffering from tinnitus at presen (☐ right ☐ left ☐ both sides)	☐ Have undergone LASIK surgery before
☐ Have suffered from ear illness before<br>Diagnosis (right left)	☐ Have suffered from eye illness before<br>Diagnosis ( )
☐ Receiving outpatient care for ear illness at present<br>Diagnosis (right left)	☐ Receiving outpatient care for eye illness at present<br>Diagnosis ( )

3. Please enter the name of each illness that you have suffered from before. If you have never suffered from any significant illness, please check the box against "None."

☐ None

Diagnosis	Age upon onset	Diagnosis	Age upon onset
	years		years
	years		years
	years		years

4. Inquiry about your family doctor and/or the medical facility at which you are receiving outpatient care

- Do you have your family doctor (attending physician)? ☐ No ☐ Yes

- Please enter the name of the medical facility that you visit, the name of the department that you visit, and the name of the doctor who attends to you.

Medical facility ( ) Specialty ( ) Doctor's name ( )

Medical facility ( ) Specialty ( ) Doctor's name ( )

Name: years old I D | | | | | | | | | | | 1 | 2

Questionnaire

5.Family history

- Do any of your blood relatives (parents, grandparents, brothers/sisters and so on) have a history of the following illnesses?

	Hypertension	Diabetes mellitus	Angina pectoris/ Myocardial infarction	Stroke	Unexplained sudden death	Glaucoma	Cancer	Please enter the cancer-affected site
Father	☐	☐	☐	☐	☐	☐	☐	Site: ( )
Mother	☐	☐	☐	☐	☐	☐	☐	Site: ( )
Grandparent	☐	☐	☐	☐	☐	☐	☐	Site: ( )
Brother/Sister	☐	☐	☐	☐	☐	☐	☐	Site: ( )

6. Were you found to have any abnormality (requiring re-test or detailed test) at a previous health checkup performed within the previous 3 years (including checkup at other facilities); if so, please mention the abnormality and the results of the re-test or detailed test.

Abnormality found	Re-test/detailed test results (please encircle the relevant alternative)				In other cases, enter the outcome in the parentheses.
( ) →	No abnormality	• Receiving outpatient care/ follow-up at hospital	• Advised to undergo follow-up through health checkups	• No visit (No test)	• ( )
( ) →	No abnormality	• Receiving outpatient care/ follow-up at hospital	• Advised to undergo follow-up through health checkups	• No visit (No test)	• ( )
( ) →	No abnormality	• Receiving outpatient care/ followup at hospital	• Advised to undergo follow-up through health checkups	• No visit (No test)	• ( )

7. Inquiry about endoscopy

- Have you undergone gastric endoscopy within the previous one year?

☐ No ☐ Yes If "Yes," please enter the endoscopy results. ( )

- Have you undergone colorectal endoscopy within the previous 2 years?

☐ No ☐ Yes If "Yes," please enter the endoscopy results. ( )

When was the colorectal endoscopy performed? (year month) Approximate or incomplete answer will also suffice.

8. Inquiry about subjective symptoms

Please check the symptoms you have now or have experienced during the previous one year.

☐ None If you have not had any of the symptoms listed below, please check the box against "None."

☐ Heartburn	☐ Frequent cough	☐ Palpitation	☐ Bleeding during evacuatio
☐ Gastric discomfor	☐ Frequent sputum	☐ Shortness of breath	☐ Pain during urinatio
☐ Stomachache	☐ Chest pain	☐ Headache	☐ Residual urine sensation
☐ Nausea	☐ Tightness of the chest	☐ Dizziness/lightheadedness	☐ Difficulty in urinatior
☐ Difficulty in swallowing	☐ Arrhythmia	☐ Malaise	☐ Loss of consciousness experienced within the previous one year

9. Please check the appropriate box describing your job. *If your job does not fall under any of those listed below, please enter your occupation in the column of "Others."

☐ Administrative position	☐ Teacher	☐ Sales	☐ Clerk	☐ Jobless/Retired upon reaching the age limit
☐ Physician	☐ Self-employed	☐ Housewife	☐ Others ( )	

Name: years old I D [ ] [ ] [ ] [ ] [ ] [ ] [ ] [ ] [ ] [ ] 1 3

Questionnaire

10. Inquiry about your lifestyle Please check the relevant alternatives or enter numeral:

- Do you smoke habitually? ☐ No, I don't smoke ☐ Yes, I smoke. ☐ I have stopped smoking. ([ ][ ] years ago)

  If you are a smoker or ex-smoker, please enter the number of cigarettes that you smoke/smoked daily and the duration of smoking. Daily [ ][ ] cigarettes ca. [ ][ ] years

- Please enter the frequency of your drinking alcohol. ☐ (1) Every day ☐ (2) Sometimes ☐ (3) Having stopped drinking ([ ][ ] years ago) ☐ Seldom (unable to drink)

  [If you have checked (1), (2) or (3):]

  Please answer the volume of alcohol consumed on a typical drinking day. ☐ Less than 1 Go ☐ 1 to less than 2 Go ☐ 2 to less than 3 Go ☐ 3 Go or more

  *Japanese wine 1 Go (180 mL) is approximately equivalent to: beer 500 mL, Shochu (25%) 110 mL, one double-glass of whisky (60 mL), two glasses of wine (240 mL)

  On how many days of the week do you drink alcohol? (If your answer is (3), on how may days of the week did you used to drink?) [ ] days

- Have you lost weight by 3 kg or more during the previous 1 year? ☐ No ☐ Yes

  If "Yes," please give the reason for the weight loss of 3 kg or more. ☐ Diet therapy or exercise ☐ Reason unknown ☐ Other reasons ( )

- Have you gained weight by 10 kg or more as compared to your weight recorded when you were 20 years old? ☐ No ☐ Yes
- Have you been exercising (for 30 minutes or more, until you sweat lightly) on 2 days or more of the week for one year or longer? ☐ No ☐ Yes
- Do you walk or engage in similar physical activity for one hour or more per day in your daily life? ☐ No ☐ Yes
- Do you walk faster than people of the same age and gender as you? ☐ No ☐ Yes
- Please select the style of eating food from the alternatives given below. Check one alternative.

  ☐ I can chew/eat any food. ☐ I can hardly chew food.

  ☐ I sometimes have difficulty in chewing food because of problems with my teeth, gums, occlusion or the like.

- How about your speed of eating as compared to other people? ☐ Faster ☐ Normal ☐ Slower
- Do you take your supper within 2 hours of your before bedtime thrice or more per week? ☐ No ☐ Yes
- Do you take snack or sweet beverage in addition to three meals (breakfast, lunch and supper)? ☐ Every day ☐ Sometimes ☐ Seldom
- Do you skip breakfast three times or more per week? ☐ No ☐ Yes
- How many days, on average, per week have you eaten fish in the past 1 month? [ ] days
- Do you intend to improve your lifestyle (exercise, dietary style, etc.)? Check one alternative.

  ☐ No intention to improve my lifestyle

  ☐ Already begun to improve my lifestyle (less than 6 months ago)

  ☐ Intend to improve my lifestyle (within the next about 6 months)

  ☐ Already begun to improve my lifestyle (since 6 months ago or more)

  ☐ Intend to improve my lifestyle soon (within about 1 month) and have begun to take small steps in that direction

- If you are given the opportunity to receive health guidance for improvement of your lifestyle, will you utilize it? ☐ No ☐ Yes
- Do you feel sufficiently refreshed after a night of sleep? ☐ No ☐ Yes
- How many hours do you sleep daily, on average? [ ] hours

Name: years old I D | | | | | | | | | | 1 4

Questionnaire

◎ Questions for women only

1. Inquiry to judge the appropriateness of conducting radiography. Please check the relevant alternative or enter a numeral.

☐ I am definitely not pregnant now. ☐ I may be pregnant now (or, I am not sure if I am pregnant). ☐ I am pregnant (☐ months of pregnancy)

*If you have answered "I may be pregnant now (or, I am not sure if I am pregnant)" or "I am pregnant," you cannot undergo any radiographic examination.

2. Inquiry about menstruation. Please check the relevant alternative.

Are you currently menstruating? ☐ No ☐ Yes

*If you are currently menstruating, it can affect the results of your urine test. We recommend that you postpone your health checkup to a time when you are not menstruating.

◎ Inquiry for those who desire to undergo a breast examination. Please check the relevant alternative.

1. Receiving outpatient care for breast disease at present ☐ No ☐ Yes Diagnosis ( )
*Receiving doctor's care through periodic visits to a hospital
2. Breastfeeding at present ☐ No ☐ Yes
3. I weaned my infant off breast milk recently (within the previous 6 months). ☐ No ☐ Yes
4. Currently receiving augmentation mammoplasty (injection, etc.) ☐ No ☐ Yes
5. I have an implanted cardiac pacemaker. ☐ No ☐ Yes
6. I have an encephalo-peritoneal shunt. ☐ No ☐ Yes

• Currently receiving treatment for breast cancer or visiting a hospital for outpatient care
• Underwent surgery for breast cancer less than 6 years ago
• Breastfeeding at present
• Recently weaned infant off breast milk (within the previous 6 months)
• Pregnant at present
• Receiving augmentation mammoplasty
Breast examination will not be performed under these circumstances, even if the subject requests it.

7. Family history: Do any of your blood relatives (grandparents, parents, brothers/sisters, children) have a history of any of the illnesses listed below?
☐ Breast cancer (Who) ☐ Ovarian cancer (Who) ☐ Prostate cancer (Who) ☐ Other cancer (Site: Who:)
8. ◎ I have undergone surgery for breast cancer. ☐ No ☐ Yes (☐ right ☐ left)
◎ I have undergone surgery for breast illness (benign). ☐ No ☐ Yes (☐ right ☐ left)

◎Inquiry for those who desire to undergo gynecologic examination. Please check the relevant alternative or enter a numeral.

*The gynecological test cannot be received during the menstruation period. If you are during menstruation, please change the schedule.

1. Currently receiving outpatient care for gynecological disease ☐ No ☐ Yes Diagnosis ( )
*Receiving doctor's care through periodic visits to a hospital
2. I have undergone gynecological surgery. ☐ No ☐ Yes Please enter the operation you have received ( )
3. I have a history of pregnancy. ☐ No ☐ Yes (Frequency of delivery ☐ times) *If you have been pregnant before, please enter your 'para' status.
4. Experience of sexual intercourse ☐ Absent ☐ Present
5. Menstrual cycle ☐ Regular ☐ Irregular ☐ Menopause (at age ☐☐)
6. Last menstrual period ☐☐ (month) ☐☐ (day), started ☐ days
7. Menstrual pain ☐ Absent ☐ Present
8. Menstrual blood loss ☐ Small ☐ Ordinary ☐ Large

*If you are postmenopausal, you may skip questions 6 through 8.
